# Neutrophil-Derived Cytokines: Facts Beyond Expression

**DOI:** 10.3389/fimmu.2014.00508

**Published:** 2014-10-21

**Authors:** Cristina Tecchio, Alessandra Micheletti, Marco A. Cassatella

**Affiliations:** ^1^Section of Hematology, Department of Medicine, School of Medicine, University of Verona, Verona, Italy; ^2^Section of General Pathology, Department of Pathology and Diagnostics, School of Medicine, University of Verona, Verona, Italy

**Keywords:** neutrophil, cytokine, chemokine, human, mouse

## Abstract

Polymorphonuclear neutrophils, besides their involvement in primary defense against infections – mainly through phagocytosis, generation of toxic molecules, release of enzymes, and formation of extracellular traps – are also becoming increasingly important for their contribution to the fine regulation in development of inflammatory and immune responses. These latter functions of neutrophils occur, in part, *via* their *de novo* production and release of a large variety of cytokines, including chemotactic cytokines (chemokines). Accordingly, the improvement in technologies for molecular and functional cell analysis, along with concomitant advances in cell purification techniques, have allowed the identification of a continuously growing list of neutrophil-derived cytokines, as well as the characterization of their biological implications *in vitro* and/or *in vivo*. This short review summarizes crucial concepts regarding the modalities of expression, release, and regulation of neutrophil-derived cytokines. It also highlights examples illustrating the potential implications of neutrophil-derived cytokines according to recent observations made in humans and/or in experimental animal models.

## Introduction

**Figure 1:**
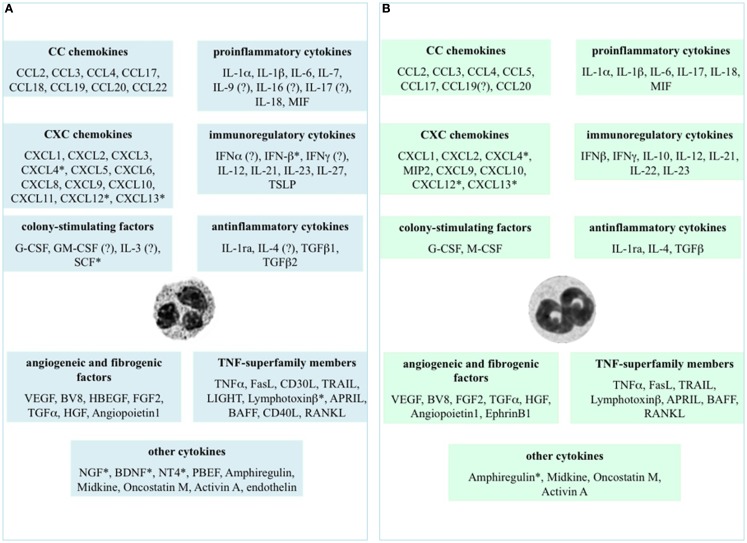
**Cytokines that neutrophils can potentially express and/or produce**. Expression and/or production of the listed cytokines have been validated in human **(A)** and murine **(B)** neutrophils by gene expression techniques, immunohistochemistry, enzyme-linked immunoadsorbent assays (ELISAs), or biological assays. *Refers to studies performed at the mRNA level only. ? Indicates controversial data.

The immune system is well suited to a quick and specific response against foreign invaders, its ultimate objective being to protect an organism from injury and disease. Cytokines represent an integral component of the signaling networks among various cells, being, for instance, essential for the development and regulation of innate and adaptive immune processes. Cytokines constitute a large family of small proteins that are produced by immune and non-immune cells and that act locally among neighboring cells to direct important biological processes such as inflammation, immunity, repair, and angiogenesis (1). In this context, the relatively recent acquisition that also activated neutrophils, among leukocytes, are able to express and release a number of cytokines (2) has convinced researchers in the field to reconsider and thus reinvestigate neutrophil biological role not only in the context of inflammatory processes, but also in other conditions (3–5). By doing so, it has clearly emerged that, given the large array of cytokines that may potentially be produced (Figure 1), neutrophils can be functionally involved either in physiological processes such as hematopoiesis, angiogenesis, and wound healing (2, 6), or in pathological processes including inflammatory, infectious, autoimmune, and neoplastic diseases (2, 4, 7, 8). Needless to say that, based on the afore-mentioned considerations, there is an increasing interest in clearly identifying and characterizing all the cytokines that neutrophils may produce, as well as their precise role in diseases, with the purpose of identifying novel targets for therapeutic interventions.

## Cytokine Expression in Neutrophils: Tools and Caveats

Our knowledge of the production of cytokines by neutrophils mostly derives from studies on humans and mice. Human neutrophils are usually isolated from the peripheral blood, while murine neutrophils are traditionally isolated from the bone marrow or the peritoneal cavity. Similarly to other cell types, also in neutrophils the production of cytokines is usually preceded by an increased accumulation of the related mRNA transcripts, which can be detected by techniques such as quantitative reverse-transcription polymerase chain reaction (qPCR), Northern Blotting, ribonuclease protection assay, and *in situ* hybridization (9). The fact that, per-cell, neutrophils possess 10–20 times less RNA than other leukocytes (10), illustrates the need for using rigorous isolation procedures to allow the recovery of highly purified cell populations. In fact, a mononuclear cell contamination of neutrophils equal to only 1% (or even less) can translate into up to 20–30% RNA contamination (9): the latter, depending on the expression levels of the cytokine mRNA under study, may obviously produce false positive results attributed to neutrophils. Nowadays, reliable tools guaranteeing the isolation of highly purified CD66b^+^/C11b^+^/CD16^−^neutrophils (e.g., by immunomagnetic negative selection) are commercially available (11, 12). Even in mice, cytokine production by neutrophils should be carefully reevaluated by utilizing Ly6G^high^/CD11b^high^ positive cells only, as recently done (13).

It is also important to mention that, at least *in vitro* and with few exceptions, neutrophils usually make fewer molecules of a given cytokine than monocytes/macrophages or lymphocytes do on a per-cell basis (2, 10). *In vivo*, however, neutrophils constitute the majority of infiltrating cells in inflamed tissues and often outnumber mononuclear leukocytes by one to two orders of magnitude. Thus, the fact that neutrophils clearly predominate over other cell types under various *in vivo* conditions suggests that, under those circumstances, the contribution of neutrophil-derived cytokines can be of foremost importance. In any case, neutrophil-derived cytokines can be measured in cell-free supernatants or in cell lysates by using various methods, including enzyme-linked immunoadsorbent assays, radioimmunoassays, immunoprecipitation after metabolic labeling, bioassays, immunohistochemistry, intracellular staining by flow cytometry, or confocal microscopy. In our opinion, the latter two techniques should be used only to support other methods, due to potential artifacts consequent to antibody cross-reactivity or elevated neutrophil autofluorescence. Another important caveat for *in vitro* studies regards the necessity to always use endotoxin-free tissue culture media or reagents, since neutrophils respond to picomolar concentrations of lipopolysaccharide (LPS) (2, 10).

The literature demonstrates that neutrophils express and produce cytokines either constitutively or upon activation by microenvironmental stimuli (2). A variety of neutrophil receptors, including colony-stimulating factor and cytokine receptors, G protein coupled-, Fcγ- and complement receptors, or many pattern recognition receptors (PRR) (germline-encoded receptors recognizing structures in microorganisms and tissue damage products), have been shown to trigger cytokine production in neutrophils (2, 14). Among PRR, human and mice neutrophils are known to express almost all Toll-like receptors (TLRs), as well as to respond to their ligands [(15), and references therein]. TLR3 and TLR7 are actually the only TLRs that human neutrophils do not express (16–18), unlike murine neutrophils that instead accumulate significantly high levels of TLR7 mRNA under inflammatory conditions (19, 20). Moreover, murine neutrophils do not basally express TLR3 transcripts (16, 21, 22) even though eventual accumulation has never been quantified under inflammatory condition. Interestingly, in human neutrophils, TLR4 activation by LPS fails to directly trigger the production of type I INFs and type I IFN-dependent genes as in other cell types (23, 24), due to its inability to engage the so called “TIR domain-containing adaptor protein inducing interferon β (TRIF)/TRIF-related adaptor molecule (TRAM)”-dependent pathway (23, 24).

Finally, an increasing number of studies have documented that TLR-induced cytokine expression by neutrophils can be positively/negatively influenced by immunomodulating factors such as IFNγ (25, 26) and IL-10 (27), respectively.

Following stimulation, neutrophils control their cytokine expression and production patterns by utilizing fine regulatory mechanisms acting at the transcriptional and/or post-transcriptional level (2, 4, 7, 28). Interestingly, recent studies have demonstrated that also microRNAs may regulate cytokine and chemokine production in neutrophils. For instance, miR9 was for the first time demonstrated to inhibit NFKB1/p50 transcripts in human neutrophils exposed to pro-inflammatory signals, operating in this manner as a feedback control for NFKB1/p50-dependent responses (29). More recently, miR-223 has been shown to negatively control the production of CXCL2, CCL3, and IL-6 by neutrophils, in a mouse model of *Mycobacterium tuberculosis* infection (30). These latter data have contributed in shedding light on the hitherto controversial role of neutrophils in tuberculosis (31), as they suggest that miR-223-dependent inhibitory effects may negatively control leukocyte chemotaxis at late stages of lung inflammation by means of developmentally accumulated miR-223 (30). Interestingly, examples of *de novo* synthesized cytokines that neutrophils store in significant amounts within intracellular pools also exist, and include B-cell activating factor (BAFF), TNF-related apoptosis-inducing ligand (TRAIL), CXCL8, CCL20, and interleukin (IL) 1 receptor antagonist (IL1-ra) (32, 33). However, very little is known on the precise intracellular localization and trafficking of these various cytokines and chemokines. Thus, much more work is needed to understand if, and how, the various neutrophil granules or other intracellular organelles contribute to cytokine metabolism and release (34).

## Cytokine Production by Neutrophils: Facts

Figure 1 displays the cytokines that, to date, have been shown to be expressed or produced by, respectively, human (panel A) and murine (panel B) neutrophils, either constitutively or upon stimulation. It is evident that neutrophils express/produce cytokines belonging to various families, mostly including pro-inflammatory/anti-inflammatory cytokines, chemokines, immunoregulatory cytokines, tumor necrosis factor (TNF) superfamily members, and angiogenic/fibrogenic factors. At first sight, an analysis of the figure immediately suggests that the ability of neutrophils to produce such a variety of cytokines enables them to significantly influence not only the multiple aspects of the inflammatory and immune responses, but also antiviral defense, hematopoiesis, angiogenesis, and fibrogenesis. Importantly, numerous *in vivo* observations have confirmed and reproduced the *in vitro* findings, as well as often clarifying their biological meanings and implications (2). It can also be noticed that, in spite of a substantial conservation between the human and murine genomes (35), some differences in cytokine production exist between the two species (Figure 1), thus warning toward a *sic et simpliciter* extrapolation of data from experimental systems in animals to humans, or *vice versa*. In the case of IL-10, for instance, basal differences in the chromatin status of the *IL-10* locus, rather than a different responsiveness to activating signals, have been shown to account for the differential ability of human and murine neutrophils to switch on transcription of the IL-10 gene (36).

In the following paragraphs, some up-to-date findings illustrating potential biological roles that neutrophil-derived chemokines, pro-inflammatory/immunoregulatory cytokines, and TNF-superfamily members might have, under pathophysiological contexts, are briefly mentioned. For an extensive description on the role of neutrophil-derived cytokines in cancer and in angiogenesis, the reader may refer to our recent reviews (6, 8).

### Chemokines

Chemokines, amongst the cytokines produced by neutrophils, are of particular relevance because of their singular ability to selectively recruit discrete cell populations into sites of injury and thereby effectively regulate leukocyte trafficking (37). In addition, chemokines play fundamental roles in coordinating immune system responses, in regulating B- and T-cell development and in modulating angiogenesis (38). As displayed in Figure 1, both human and murine neutrophils may potentially produce several chemokines upon activation, including IL-8/CXCL8, GROα/CXCL1, MIG/CXCL9, IP-10/CXCL10, and I-TAC/CXCL11, monocyte chemotactic protein-1 (MCP-1/CCL2), macrophage inflammatory protein-1α (MIP-1α/CCL3) and MIP-1β/CCL4 (37). Because the chemokines produced by neutrophils are primarily chemotactic for neutrophils, monocytes, dendritic cells (DCs), natural killer (NK) cells, and T-helper type 1 (Th1) and type 17 (Th17) cells, a potential role for neutrophils in amplifying their own arrival (39), as well as in orchestrating the sequential recruitment to, and activation of, distinct leukocyte types in the inflamed tissue, is plausible (37, 40). And in fact, in *in vitro* experiments, it has been demonstrated that: (i) human neutrophils activated by neutrophil-activating protein A from *Borrelia burgdorferi* recruit IFNγ- and IL-17-producing T lymphocytes *via* CCL2, CCL20, and CXCL10 release (41); (ii) LPS-activated neutrophils induce chemotaxis of immature and mature DCs, as well as adhesion of CCR6- and CCR7-expressing T cells *via* CCL19 and CCL20 (42); and (iii) IFNγ plus LPS-activated neutrophils induce chemotaxis of Th17 cells through CCL2 and CCL20 (11). Moreover, in different mouse models it has been proved that: (i) DCs are recruited to the *Leishmania* inoculation site by neutrophil-derived CCL3 (43); (ii) immature DCs are strongly attracted by neutrophil-derived CCL3, CCL4, CCL5, and CCL20 triggered by *Toxoplasma gondii* (44); and (iii) macrophage influx to granulomas is dependent on CCL3 and CCL4 released by activated neutrophils (45). More recently, CCL17, a chemokine that binds to CCR4, a chemokine receptor expressed in T-helper type 2 (Th2) cells and in regulatory T cells (Tregs) (46), has been added to the list of neutrophil-derived chemokines (47, 48). Consistently, tumor-associated neutrophils (TANs) have been shown to chemoattractants Tregs in a mouse model of cancer, mainly *vi*a CCL17 (49). Because neutrophil depletion, in this model, was shown to reduce Tregs recruitment and, consequently, tumor growth, data provide, for the first time, a clear link between TANs and Tregs, acting together to impair antitumor immunity (49).

### Pro-inflammatory and immunoregulatory cytokines

As shown in Figure 1, neutrophils may become a significant source of pro-inflammatory and/or immunoregulatory cytokines. Among these cytokines, recent research has focused on neutrophil-derived IL-17 and IFNγ, as well as on their eventual role in inflammatory diseases and/or in protection against infections. However, since the data on the effective capacity of human neutrophils to express/produce IL-17 (11, 50) or IFNγ (51, 52) are still controversial in literature, we can only report on studies carried out in mice.

For instance, IL-23- and, indirectly, IL-12-, activated Gr-1^+^-neutrophils, but not Th17 cells, have been found to be the predominant source of IL-17A in a mouse model of kidney ischemia-reperfusion injury (IRI) (53). Such neutrophil-derived IL-17 has been then shown to regulate natural killer T cells (NKT) activation, IFNγ production, neutrophil infiltration, ultimately inflammation, and tissue injury (53), thus establishing its requirement for kidney injury following IRI. In another, more recent study, researchers have identified a murine population of bone marrow neutrophils that constitutively express the transcription factor RORγt, and that rapidly produce and respond to IL-17A in a IL-23 plus IL-6-dependent manner (54). Autocrine activity of IL-17A on such neutrophil subset has been shown not only to induce the production of reactive oxygen species (ROS), but also to increase neutrophil-mediated fungal killing *in vitro* and, importantly, also in an *in vivo* model of *Aspergillus fumigatus*-induced keratitis (55). In a similar fashion, IL-17A produced by Ly6G^+^-neutrophils, but not by NKT or γδT cells, was found to be important in providing protection against early pneumonic plague infection in mice (56). In this model, however, neutrophil-derived IL-17A did not significantly change neutrophil bactericidal activities, but was instead crucial for the IFNγ-mediated programming of M1 pro-inflammatory macrophages after *Yersinia pestis* challenge (56).

A number of *in vivo* experiments have reported that, in response to a variety of pathogens, including *Nocardia asteroides* (57), *Listeria monocytogenes* (58), and *Plasmodium berghei* (59), murine neutrophils secrete IFNγ, a crucial orchestrator for host defense against intracellular pathogen. Neutrophils have been found to be an important source of IFNγ also upon *Toxoplasma gondii* infection, in a model of genetically modified mice lacking all lymphoid cells due to deficiencies in Recombination Activating Gene 2 (RAG2) and IL-2Rγc genes (60). In these mice, although insufficient for complete host protection, neutrophil-derived IFNγ was found to be TLR11-independent and to significantly reduce pathogen load therefore extending mice survival (60). Moreover, other studies have shown that migrated neutrophils are responsible for the early production of IFNγ during pneumonia infections, in turn regulating bacterial clearance in mice (61). Interestingly, such IFNγ production does not require either IL-12, or CD11/18 complex, CD44, TLR2, TLR4, TRIF, and Nrf2, while it is nearly abolished in Nox2 deficient mice (62). Altogether, data not only underline the complexity of the neutrophil responses during pneumonia, but also highlight how tightly regulated is the process of IFNγ induction in neutrophils, as it likely involves interactions between multiple signaling pathways.

### TNF-superfamily members

Human and murine neutrophils also express and produce many TNF-superfamily members (Figure 1A,B), although at variable levels (2). For example, human neutrophils synthesize – at least *in vitro –* very low amounts of TNFα (in the order of picogram per milliliter per million cells) in response to TLR agonists (2), which nonetheless exert potent autocrine effects in amplifying neutrophil-derived cytokines and chemokines [(63) and other unpublished observations from our group]. *In vivo*, neutrophil-derived TNFα has been recently described to either instruct skin Langerhans cells to prime antiviral immune responses (64), or to stimulate melanoma cells to migrate towards endothelial cells and metastasize to the lungs, in a mouse model of primary cutaneous melanoma undergoing repetitive ultraviolet (UV) exposure (65). Moreover, through application of confocal intravital microscopy to the mouse cremaster muscle, it has been very recently shown that chemoattractans-responding neutrophils release TNFα when in close proximity of endothelial cell junctions. Further, in TNF receptor (TNFR) (−/−) mice, neutrophils accumulated normally in response to chemoattractants administered to the cremaster muscle or dorsal skin, whereas neutrophil-dependent plasma protein leakage was abolished, suggesting that neutrophil-derived TNFα mediates microvascular leakage (66).

On the other hand, neutrophils have turned-out as a major source of both BAFF (67) and a proliferation inducing ligand (APRIL) (68), two TNF members that are critical for B-cell maturation, function, and survival. Accordingly, BAFF and APRIL (other than IL-21 and, possibly, CD40L) have proven to be fundamental mediators of the functions of a recently identified neutrophil subset in human spleen – the so called “B-helper” neutrophil subset – precisely for their ability to stimulate immunoglobulin diversification and production by splenic marginal zone B-cells (69). An involvement of BAFF-producing splenic neutrophils in the pathogenesis of murine lupus has been also demonstrated in a recent study, suggesting that neutrophils help to shape CD4^+^ T cell responses *via* BAFF, which in turn contributes to the production of pathogenic autoantibodies (70).

Finally, another TNF-superfamily member that both human and murine activated neutrophils can produce and release is TRAIL, a trans-membrane/soluble molecule involved in tumor cell killing and autoimmunity (33). In humans, neutrophil- derived TRAIL has been classically detected *ex vivo* in the context of intravesical BCG infusion and systemic IFNα administration to treat, respectively, bladder cancer and chronic myeloid leukemia (71, 72). Since previous *in vitro* data had shown an effective TRAIL-mediated cytotoxicity of neutrophils towards leukemic cells (73, 74), it is conceivable to hypothesize further studies aimed at harnessing neutrophils against tumors, possibly *via* TRAIL induction. Nonetheless, our knowledge on the TRAIL production by neutrophils has been more recently extended to the mouse system, as neutrophil-derived TRAIL has been shown to exert antiviral activities in a model of cytomegalovirus infection (75), as well as to mediate early bacterial killing in a model of pneumococcal pneumonia (76). Based on the afore-mentioned and other (2, 8, 42, 43) observations, it appears that TNF-superfamily members may contribute to a large extent to unexpected functions that neutrophils may exert.

## Concluding Remarks

During the past decades, novel functions in homeostasis and pathology have emerged for neutrophils, mainly for their ability to represent a source for a variety of cytokines. It is plausible that with the development of very efficient cell isolation techniques and the increased availability of neutrophils purified from various compartments, such as spleen, peritoneal exudates, lungs, oral cavity, skin, bone marrow, cord blood, and placenta, our knowledge of the repertoire of cytokines produced by human and mouse neutrophils will expand.

Apart from what has been briefly summarized in this review, a number of issues remain to be better explored and/or clarified in this research area.

For instance, we need to elucidate all the stimuli that are able to induce cytokine synthesis in neutrophils. Such studies would be particularly helpful in understanding the pathogenesis of diseases in which neutrophils represent (or are presumed to be) the first cell type encountering, and interacting with, the etiologic agent. In fact, we know that the interaction of neutrophils with a given agonist produces a characteristic, stimulus-specific response (2). The recent findings that human neutrophils possess intracellular sensor systems that allow the recognition of foreign and potentially dangerous RNA and DNA, as well as the inflammasomes (17, 77–80), demonstrates that neutrophils, *via* cytokine/chemokine release, are in the position to act at the front-line of immunity not only toward extracellular, but also toward intracellular microorganisms, including viruses. Another aspect that needs to be more systematically dissected concerns the identification of the molecular mechanisms controlling cytokine expression in neutrophils. Such studies may lead to the identification of novel, maybe neutrophil-specific, transcription factors, or of neutrophil-specific chromatin organization programs (36). Finally, more information on the *in vivo* role of neutrophil-derived cytokines should be acquired in humans, since it mostly derives from experimental animal models. By doing so, it is tempting to predict that unanticipated functions of neutrophils can be discovered. Future challenges for scientists in the field will be to translate all these new insights into efficacious neutrophil-targeted therapies for the treatment of inflammatory conditions without compromising immunity.

## Conflict of Interest Statement

The authors declare that the research was conducted in the absence of any commercial or financial relationships that could be construed as a potential conflict of interest.

## Abbreviations

APRIL, a proliferation inducing ligand; BAFF, B-cell activating factor; BNDF, brain-derived neurotrophic factor; Bv8, bombina variegata peptide 8; HB-EGF, heparin-binding endothelial growth factor, (EGF)-like growth factor; FGF2, fibroblast growth factor-2; G-CSF, granulocyte colony-stimulating factor; GM-CSF, granulocyte/macrophage-colony-stimulating factor; HGF, hepatocyte growth factor; IL1-ra, interleukin 1 receptor antagonist; LIGHT, lymphotoxin-related inducible ligand that competes for glycoprotein D binding to HVEM on T cells; M-CSF, macrophage-colony-stimulating factor; MIF, macrophage migration inhibitory factor; MIP2, macrophage inflammatory protein 2; NGF, nerve growth factor; NT4, neurotrophin 4; PBEF, pre-B-cell colony enhancing factor; RANKL, receptor activator of nuclear factor kappa-B (NFκB) ligand; SCF, stem cell factor; TGFα, transforming growth factor alpha; TGFβ, transforming growth factor beta; TRAIL, TNF-related apoptosis-inducing ligand; TSLP, thymic stromal lymphopoietin; VEGF, vascular endothelial growth factor.

## References

[1] Feldmann M. Many cytokines are very useful therapeutic targets in disease. J Clin Invest (2008) 118:3533–6. 10.1172/JCI37346 18982159 PMC2575703

[2] Cassatella MA. Neutrophil-derived proteins: selling cytokines by the pound. Adv Immunol (1999) 73:369–509. 10.1016/S0065-2776(08)60791-9 10399011

[3] Mócsai A. Diverse novel functions of neutrophils in immunity, inflammation, and beyond. J Exp Med (2013) 210:1283–1129. 10.1084/jem.20122220 23825232 PMC3698517

[4] Jaillon S Galdiero MR Del Prete D Cassatella MA Garlanda C Mantovani A. Neutrophils in innate and adaptive immunity. Semin Immunopathol (2013) 35:377–94. 10.1007/s00281-013-0374-8 23553214

[5] Mayadas TN Cullere X Lowell CA. The multifaceted functions of neutrophils. Annu Rev Pathol (2014) 9:181–218. 10.1146/annurev-pathol-020712-164023 24050624 PMC4277181

[6] Tecchio C Cassatella MA. Neutrophil-derived cytokines involved in physiological and pathological angiogenesis. Chem Immunol Allergy (2014) 99:123–37. 10.1159/000353358 24217606

[7] Mantovani A Cassatella MA Costantini C Jaillon S. Neutrophils in the activation and regulation of innate and adaptive immunity. Nat Rev Immunol (2011) 11:519–31. 10.1038/nri3024 21785456

[8] Tecchio C Scapini P Pizzolo G Cassatella MA. On the cytokines produced by human neutrophils in tumors. Semin Cancer Biol (2013) 23:159–70. 10.1016/j.semcancer.2013.02.004 23410636

[9] Tamassia N Cassatella MA Bazzoni F. Fast and accurate quantitative analysis of cytokine gene expression in human neutrophils. Methods Mol Biol (2014) 1124:451–67. 10.1007/978-1-62703-845-4_27 24504968

[10] Scapini P Calzetti F Cassatella MA. On the detection of neutrophil-derived vascular endothelial growth factor (VEGF). J Immunol Methods (1999) 232:121–9. 10.1016/S0022-1759(99)00170-2 10618514

[11] Pelletier M Maggi L Micheletti A Lazzeri E Tamassia N Costantini C Evidence for a cross-talk between human neutrophils and Th17 cells. Blood (2010) 115:335–43. 10.1182/blood-2009-04-216085 19890092

[12] Davey MS Tamassia N Rossato M Bazzoni F Calzetti F Bruderek K Failure to detect production of IL-10 by activated human neutrophils. Nat Immunol (2011) 12:1017–8. 10.1038/ni.2111 22012430

[13] Zhang X Majlessi L Deriaud E Leclerc C Lo-Man R. Coactivation of Syk kinase and MyD88 adaptor protein pathways by bacteria promotes regulatory properties of neutrophils. Immunity (2009) 31:761–71. 10.1016/j.immuni.2009.09.016 19913447

[14] Benelli R Morini M Carrozzino F Ferrari N Minghelli S Santi L Neutrophils as a key cellular target for angiostatin: implications for regulation of angiogenesis and inflammation. FASEB J (2002) 16:267–9. 11772950 10.1096/fj.01-0651fje

[15] Thomas CJ Schroder K. Pattern recognition receptor function in neutrophils. Trends Immunol (2013) 34:317–28. 10.1016/j.it.2013.02.008 23540649

[16] Hayashi F Means TK Luster AD. Toll-like receptors stimulate human neutrophil function. Blood (2003) 102:2660–9. 10.1182/blood-2003-04-1078 12829592

[17] Tamassia N Le Moigne V Rossato M Donini M McCartney S Calzetti F Activation of an immunoregulatory and antiviral gene expression program in poly(I:C)-transfected human neutrophils. J Immunol (2008) 181:6563–73. 10.4049/jimmunol.181.9.6563 18941247

[18] Janke M Poth J Wimmenauer V Giese T Coch C Barchet W Selective and direct activation of human neutrophils but not eosinophils by Toll-like receptor 8. J Allergy Clin Immunol (2009) 123:1026–33. 10.1016/j.jaci.2009.02.015 19361845

[19] Charmoy M Megnekou R Allenbach C Zweifel C Perez C Monnat K Leishmania major induces distinct neutrophil phenotypes in mice that are resistant or susceptible to infection. J Leukoc Biol (2007) 82:288–99. 10.1189/jlb.0706440 17449725

[20] Wang JP Bowen GN Padden C Cerny A Finberg RW Newburger PE Toll-like receptor-mediated activation of neutrophils by influenza A virus. Blood (2008) 112:2028–34. 10.1182/blood-2008-01-132860 18544685 PMC2518904

[21] Matsushima H Geng S Lu R Okamoto T Yao Y Mayuzumi N Neutrophil differentiation into a unique hybrid population exhibiting dual phenotype and functionality of neutrophils and dendritic cells. Blood (2013) 121:1677–89. 10.1182/blood-2012-07-445189 23305731 PMC3591793

[22] Tsuda Y Takahashi H Kobayashi M Hanafusa T Herndon DN Suzuki F. Three different neutrophil subsets exhibited in mice with different susceptibilities to infection by methicillin-resistant *Staphylococcus aureus*. Immunity (2004) 21:215–26. 10.1016/j.immuni.2004.07.006 15308102

[23] Tamassia N Le Moigne V Calzetti F Donini M Gasperini S Ear T The MyD88-independent pathway is not mobilized in human neutrophils stimulated via TLR4. J Immunol (2007) 178:7344–56. 10.4049/jimmunol.178.11.7344 17513785

[24] van Bruggen R Drewniak A Tool AT Jansen M van Houdt M Geissler J Toll-like receptor responses in IRAK-4-deficient neutrophils. J Innate Immun (2010) 2:280–7. 10.1159/000268288 20375545

[25] Cassatella MA Guasparri I Ceska M Bazzoni F Rossi F. Interferon-gamma inhibits interleukin-8 production by human polymorphonuclear leucocytes. Immunology (1993) 78:177–84. 8473010 PMC1421822

[26] Meda L Gasperini S Ceska M Cassatella MA. Modulation of proinflammatory cytokine release from human polymorphonuclear leukocytes by gamma interferon. Cell Immunol (1994) 157:448–61. 10.1006/cimm.1994.1241 8069926

[27] Bazzoni F Tamassia N Rossato M Cassatella MA. Understanding the molecular mechanisms of the multifaceted IL-10-mediated anti-inflammatory response: lessons from neutrophils. Eur J Immunol (2010) 40:2360–8. 10.1002/eji.200940294 20549669

[28] Cassatella MA. Cytokines Produced by Polymorphonuclear Neutrophils: Molecular and Biological Aspects. Berlin, Heidelberg, New York: Springer, Landes Co., (1996).

[29] Bazzoni F Rossato M Fabbri M Gaudiosi D Mirolo M Mori L Induction and regulatory function of miR-9 in human monocytes and neutrophils exposed to proinflammatory signals. Proc Natl Acad Sci U S A (2009) 106:5282–2587. 10.1073/pnas.0810909106 19289835 PMC2664036

[30] Dorhoi A Iannaccone M Farinacci M Faé KC Schreiber J Moura-Alves P MicroRNA-223 controls susceptibility to tuberculosis by regulating lung neutrophil recruitment. J Clin Invest (2013) 123:4836–48. 10.1172/JCI67604 24084739 PMC3809781

[31] Lowe DM Redford PS Wilkinson RJ O’Garra A Martineau AR. Neutrophils in tuberculosis: friend or foe? Trends Immunol (2012) 33:14–25. 10.1016/j.it.2011.10.003 22094048

[32] Scapini P Bazzoni F Cassatella MA. Regulation of B-cell-activating factor (BAFF)/B lymphocyte stimulator (BLyS) expression in human neutrophils. Immunol Lett (2008) 116:1–6. 10.1016/j.imlet.2007.11.009 18155301

[33] Cassatella MA. On the production of TNF-related apoptosis-inducing ligand (TRAIL/Apo-2L) by human neutrophils. J Leukoc Biol (2006) 79:1140–9. 10.1189/jlb.1005558 16574768

[34] Lacy P Stow JL. Cytokine release from innate immune cells: association with diverse membrane trafficking pathways. Blood (2011) 118:9–18. 10.1182/blood-2010-08-265892 21562044

[35] Mestas J Hughes CC. Of mice and not men: differences between mouse and human immunology. J Immunol (2004) 172:2731–8. 10.4049/jimmunol.172.5.2731 14978070

[36] Tamassia N Zimmermann M Castellucci M Ostuni R Bruderek K Schilling B Cutting edge: an inactive chromatin configuration at the IL-10 locus in human neutrophils. J Immunol (2013) 190:1921–5. 10.4049/jimmunol.1203022 23355741

[37] Scapini P Lapinet-Vera JA Gasperini S Calzetti F Bazzoni F Cassatella MA. The neutrophil as a cellular source of chemokines. Immunol Rev (2000) 177:195–203. 10.1034/j.1600-065X.2000.17706.x 11138776

[38] Zlotnik A Yoshie O. The chemokine superfamily revisited. Immunity (2012) 36:705–16. 10.1016/j.immuni.2012.05.008 22633458 PMC3396424

[39] Sadik CD Kim ND Luster AD. Neutrophils cascading their way to inflammation. Trends Immunol (2011) 32:452–60. 10.1016/j.it.2011.06.008 21839682 PMC3470857

[40] Scapini P Cassatella MA. Social networking of human neutrophils within the immune system. Blood (2014) 124:710–9. 10.1182/blood-2014-03-453217 24923297

[41] Codolo G Bossi F Durigutto P Bella CD Fischetti F Amedei A Orchestration of inflammation and adaptive immunity in *Borrelia burgdorferi*-induced arthritis by neutrophil-activating protein A. Arthritis Rheum (2013) 65:1232–42. 10.1002/art.37875 23371320

[42] Scapini P Laudanna C Pinardi C Allavena P Mantovani A Sozzani S Neutrophils produce biologically active macrophage inflammatory protein-3alpha (MIP-3alpha)/CCL20 and MIP-3beta/CCL19. Eur J Immunol (2001) 31:1981–8. 11449350 10.1002/1521-4141(200107)31:7<1981::aid-immu1981>3.0.co;2-x

[43] Charmoy M Brunner-Agten S Aebischer D Auderset F Launois P Milon G Neutrophil-derived CCL3 is essential for the rapid recruitment of dendritic cells to the site of *Leishmania major* inoculation in resistant mice. PLoS Pathog (2010) 6:e1000755. 10.1371/journal.ppat.1000755 20140197 PMC2816696

[44] Bennouna S Bliss SK Curiel TJ Denkers EY. Cross-talk in the innate immune system: neutrophils instruct recruitment and activation of dendritic cells during microbial infection. J Immunol (2003) 171:6052–8. 10.4049/jimmunol.171.11.6052 14634118

[45] von Stebut E Metz M Milon G Knop J Maurer M. Early macrophage influx to sites of cutaneous granuloma formation is dependent on MIP-1alpha/beta released from neutrophils recruited by mast cell-derived TNFalpha. Blood (2003) 101:210–5. 10.1182/blood-2002-03-0921 12393677

[46] Hagemann UB Gunnarsson L Géraudie S Scheffler U Griep RA Reiersen H Fully human antagonistic antibodies against CCR4 potently inhibit cell signaling and chemotaxis. PLoS One (2014) 9:e103776. 10.1371/journal.pone.0103776 25080123 PMC4117600

[47] Adib-Conquy M Pedron T Petit-Bertron AF Tabary O Corvol H Jacquot J Neutrophils in cystic fibrosis display a distinct gene expression pattern. Mol Med (2008) 14:36–44. 10.2119/2007-00081.Adib-Conquy 18026571 PMC2078559

[48] Tsuda Y Fukui H Asai A Fukunishi S Miyaji K Fujiwara S An immunosuppressive subtype of neutrophils identified in patients with hepatocellular carcinoma. J Clin Biochem Nutr (2012) 51:204–12. 10.3164/jcbn.12-32 23170048 PMC3491245

[49] Mishalian I Bayuh R Eruslanov E Michaeli J Levy L Zolotarov L Neutrophils recruit regulatory T-cells into tumors via secretion of CCL17 – a new mechanism of impaired antitumor immunity. Int J Cancer (2014) 135:1178–86. 10.1002/ijc.28770 24501019

[50] Hoshino A Nagao T Nagi-Miura N Ohno N Yasuhara M Yamamoto K MPO-ANCA induces IL-17 production by activated neutrophils in vitro via classical complement pathway-dependent manner. J Autoimmun (2008) 31:79–89. 10.1016/j.jaut.2008.03.006 18501296

[51] Ethuin F Gérard B Benna JE Boutten A Gougereot-Pocidalo MA Jacob L Human neutrophils produce interferon gamma upon stimulation by interleukin-12. Lab Invest (2004) 84:1363–71. 10.1038/labinvest.3700148 15220936

[52] Pelletier M Micheletti A Cassatella MA. Modulation of human neutrophil survival and antigen expression by activated CD4+ and CD8+ T cells. J Leukoc Biol (2010) 88:1163–70. 10.1189/jlb.0310172 20686115

[53] Li L Huang L Vergis AL Ye H Bajwa A Narayan V IL-17 produced by neutrophils regulates IFN-gamma-mediated neutrophil migration in mouse kidney ischemia-reperfusion injury. J Clin Invest (2010) 120:331–42. 10.1172/JCI38702 20038794 PMC2798679

[54] Taylor PR Roy S Leal SM Jr Sun Y Howell SJ Cobb BA Activation of neutrophils by autocrine IL-17A-IL-17RC interactions during fungal infection is regulated by IL-6, IL-23, RORγt and dectin-2. Nat Immunol (2014) 15:143–51. 10.1038/ni.2797 24362892 PMC3972892

[55] Taylor PR Leal SM Jr Sun Y Pearlman E. *Aspergillus* and *Fusarium* corneal infections are regulated by Th17 cells and IL-17-producing neutrophils. J Immunol (2014) 192:3319–27. 10.4049/jimmunol.1302235 24591369 PMC4020181

[56] Bi Y Zhou J Yang H Wang X Zhang X Wang Q IL-17A produced by neutrophils protects against pneumonic plague through orchestrating IFN-γ-activated macrophage programming. J Immunol (2014) 192:704–13. 10.4049/jimmunol.1301687 24337746

[57] Ellis TN Beaman BL. Murine polymorphonuclear neutrophils produce interferon-gamma in response to pulmonary infection with *Nocardia asteroides*. J Leukoc Biol (2002) 72:373–81. 12149429

[58] Yin J Ferguson TA. Identification of an IFN-gamma-producing neutrophil early in the response to Listeria monocytogenes. J Immunol (2009) 182:7069–73. 10.4049/Jimmunol.0802410 19454704 PMC2685461

[59] Chen L Sendo F. Cytokine and chemokine mRNA expression in neutrophils from CBA/NSlc mice infected with *Plasmodium berghei* ANKA that induces experimental cerebral malaria. Parasitol Int (2001) 50:139–43. 10.1016/S1383-5769(01)00063-0 11438437

[60] Sturge CR Benson A Raetz M Wilhelm CL Mirpuri J Vitetta ES TLR-independent neutrophil-derived IFN-γ is important for host resistance to intracellular pathogens. Proc Natl Acad Sci U S A (2013) 110:10711–6. 10.1073/pnas.1307868110 23754402 PMC3696766

[61] Yamada M Gomez JC Chugh PE Lowell CA Dinauer MC Dittmer DP Interferon-γ production by neutrophils during bacterial pneumonia in mice. Am J Respir Crit Care Med (2011) 183:1391–401. 10.1164/rccm.201004-0592OC 21169470 PMC3114063

[62] Gomez JC Yamada M Martin JR Dang H Brickey WJ Bergmeier W Mechanisms of IFN-γ Production by Neutrophils and Its Function during *S. pneumoniae* Pneumonia. Am J Respir Cell Mol Biol (2014). 10.1165/rcmb.2013-0316OC 25100610 PMC4370257

[63] Cassatella MA Meda L Bonora S Ceska M Constantin G. Interleukin 10 (IL-10) inhibits the release of proinflammatory cytokines from human polymorphonuclear leukocytes. Evidence for an autocrine role of tumor necrosis factor and IL-1 beta in mediating the production of IL-8 triggered by lipopolysaccharide. J Exp Med (1993) 178:2207–11. 10.1084/jem.178.6.2207 8245792 PMC2191270

[64] Epaulard O Adam L Poux C Zurawski G Salabert N Rosenbaum P Macrophage- and neutrophil-derived TNF-α instructs skin Langerhans cells to prime antiviral immune responses. J Immunol (2014) 193:2416–26. 10.4049/jimmunol.1303339 25057007 PMC4134993

[65] Bald T Quast T Landsberg J Rogava M Glodde N Lopez-Ramos D Ultraviolet-radiation-induced inflammation promotes angiotropism and metastasis in melanoma. Nature (2014) 507:109–13. 10.1038/nature13111 24572365

[66] Finsterbusch M Voisin MB Beyrau M Williams TJ Nourshargh S. Neutrophils recruited by chemoattractants in vivo induce microvascular plasma protein leakage through secretion of TNF. J Exp Med (2014) 211:1307–14. 10.1084/jem.20132413 24913232 PMC4076577

[67] Scapini P Nardelli B Nadali G Calzetti F Pizzolo G Montecucco C G-CSF-stimulated neutrophils are a prominent source of functional BLyS. J Exp Med (2003) 197:297–302. 10.1084/jem.20021343 12566413 PMC2193843

[68] Mhawech-Fauceglia P Kaya G Sauter G McKee T Donze O Schwaller J The source of APRIL up-regulation in human solid tumor lesions. J Leukoc Biol (2006) 80:697–704. 10.1189/jlb.1105655 16793914

[69] Puga I Cols M Barra CM He B Cassis L Gentile M B cell-helper neutrophils stimulate the diversification and production of immunoglobulin in the marginal zone of the spleen. Nat Immunol (2011) 13:170–80. 10.1038/ni.2194 22197976 PMC3262910

[70] Coquery CM Wade NS Loo WM Kinchen JM Cox KM Jiang C Neutrophils contribute to excess serum BAFF levels and promote CD4+ T cell and B cell responses in lupus-prone mice. PLoS One (2014) 9:e102284. 10.1371/journal.pone.0102284 25010693 PMC4092127

[71] Brincks EL Risk MC Griffith TS. PMN and anti-tumor immunity – the case of bladder cancer immunotherapy. Semin Cancer Biol (2013) 23:183–9. 10.1016/j.semcancer.2013.02.002 23410637

[72] Tanaka H Ito T Kyo T Kimura A. Treatment with IFNalpha in vivo up-regulates serum-soluble TNF-related apoptosis inducing ligand (sTRAIL) levels and TRAIL mRNA expressions in neutrophils in chronic myelogenous leukemia patients. Eur J Haematol (2007) 78:389–98. 10.1111/j.1600-0609.2007.00834.x 17432976

[73] Koga Y Matsuzaki A Suminoe A Hattori H Hara T. Neutrophil-derived TNF-related apoptosis-inducing ligand (TRAIL): a novel mechanism of antitumor effect by neutrophils. Cancer Res (2004) 64:1037–43. 10.1158/0008-5472.CAN-03-1808 14871835

[74] Tecchio C Huber V Scapini P Calzetti F Margotto D Todeschini G IFNalpha-stimulated neutrophils and monocytes release a soluble form of TNF-related apoptosis-inducing ligand (TRAIL/Apo-2 ligand) displaying apoptotic activity on leukemic cells. Blood (2004) 103:3837–44. 10.1182/blood-2003-08-2806 14726404

[75] Stacey MA Marsden M Pham NTA Clare S Dolton G Stack G Neutrophils recruited by IL-22 in peripheral tissues function as TRAIL-dependent antiviral effectors against MCMV. Cell Host Microbe (2014) 15:471–83. 10.1016/j.chom.2014.03.003 24721575 PMC3989063

[76] Steinwede K Henken S Bohling J Maus R Ueberberg B Brumshagen C TNF-related apoptosis-inducing ligand (TRAIL) exerts therapeutic efficacy for the treatment of pneumococcal pneumonia in mice. J Exp Med (2012) 209:1937–52. 10.1084/jem.20120983 23071253 PMC3478925

[77] Tamassia N Bazzoni F Le Moigne V Calzetti F Masala C Grisendi G IFN-β expression is directly activated in human neutrophils transfected with plasmid DNA and is further increased via TLR-4-mediated signaling. J Immunol (2012) 189:1500–9. 10.4049/jimmunol.1102985 22730532

[78] Berger M Hsieh CY Bakele M Marcos V Rieber N Kormann M Neutrophils express distinct RNA receptors in a non-canonical way. J Biol Chem (2012) 287:19409–17. 10.1074/jbc.M112.353557 22532562 PMC3365979

[79] Tamassia N Cassatella MA. Cytoplasmic receptors recognizing nucleic acids and mediating immune functions in neutrophils. Curr Opin Pharmacol (2013) 13:547–54. 10.1016/j.coph.2013.05.003 23725881

[80] Bakele M Joos M Burdi S Allgaier N Pöschel S Fehrenbacher B Localization and functionality of the inflammasome in neutrophils. J Biol Chem (2014) 289:5320–9. 10.1074/jbc.M113.505636 24398679 PMC3931087

